# Obesity, low levels of physical activity and smoking present opportunities for primary care asthma interventions: an analysis of baseline data from The Asthma Tools Study

**DOI:** 10.1038/npjpcrm.2015.58

**Published:** 2015-10-01

**Authors:** Barbara P Yawn, Matthew A Rank, Susan L Bertram, Peter C Wollan

**Affiliations:** 1 Department of Research, Olmsted Medical Center, Rochester, MN, USA; 2 Division of Allergy, Asthma and Clinical Immunology, Mayo Clinic, Scottsdale, AZ, USA; 3 Department of Research, Olmsted Medical Center, Rochester, MN, USA

## Abstract

**Background:**

Asthma prevalence, severity and outcomes are associated with various patient characteristics and lifestyle choices.

**Aims:**

To identify potentially modifiable factors associated with poor asthma outcomes among US primary care patients.

**Methods:**

Using baseline data from the Asthma Tools Study, we calculated cross-sectional frequencies of activity levels, smoking, secondhand smoke exposure and the presence of obesity, as well as rates of out-of-control asthma and asthma exacerbations. Frequencies were stratified by sex, and into three age groups: 5–11 years, 12–18 years and 19 years and older. Logistic regression was used to identify factors associated with each of the asthma outcomes.

**Results:**

In the 901 individuals enrolled in this asthma study, tobacco smoke exposure, obesity, low activity levels, poverty, inadequately controlled asthma and high asthma-related health-care utilisation were common. Across all age groups, obesity was associated with poorer asthma outcomes: either poor asthma control (odds ratio (OR)=2.3, 95% confidence interval (CI) 1.1–4.7 in 5- to 11-year-olds and OR=1.5, 95% CI 1.1–2.2 in adults) or asthma exacerbations (OR 2.9, 95% CI 1.6–5.1 in 12- to 18-year-olds and OR 1.7, 95% CI 1.1–2.5 in adults). Among adults, smoking was associated with both measures of poorer asthma outcomes; inadequate asthma control (OR=2.3, 95% CI 1.5–3.5), and asthma exacerbations (OR 1.7, 95% CI 1.1–2.6), and low physical activity were associated with poor asthma control (OR=1.5, 95% CI 1.1–2.2).

**Conclusions:**

Obesity, low levels of physical activity and smoking are common, and they are associated with poor asthma outcomes in a sample of primary care patients, suggesting important targets for intervention.

## Introduction

Asthma is common among US children and adults, with up to 1 in 8–11 children and 1 in 13 adults having received a physician diagnosis of asthma.^1,2!^ Asthma continues to be associated with a significant burden to patients, families and health-care systems.^3–6^ That burden has been shown to be increased in certain age, sex, race/ethnicity and family income groups.^7–11^ These commonly enumerated factors are seldom amenable to medical interventions.

However, asthma prevalence, severity and outcomes are also associated with several potentially modifiable patient characteristics and lifestyle choices including level of obesity,^12–14^ smoking status,^15^ levels of physical activity^16^ and exposure to secondhand smoke.^8,17–20^ Primary care physicians and practices provide the majority of asthma care^5^ and are therefore appropriate sites in which to assess the frequency of the additional potentially modifiable characteristics and lifestyle choices, highlighting opportunities to use nonmedication-based interventions to improve asthma outcomes.

This study uses the baseline data from a large pragmatic trial (The Asthma Tools Study)^21^ designed to assess the impact of introducing an asthma management tool (the Asthma APGAR system) into primary care practices. Here we present information on baseline demographic and behavioural characteristics of the enrolled patients and compare those with asthma outcomes at and in the year before enrollment. The goal is to identify potentially modifiable behavioural characteristics that might improve asthma outcomes.

## Materials and methods

The overall description of the Asthma Tools Study has been published previously.^21^ In summary, the Asthma Tools Study is a 5-year pragmatic trial comparing Asthma APGAR system–guided asthma management with usual care in family medicine and paediatric community practices. Patient enrollment occurred between February 2011 and February 2014, and baseline data for each patient were collected from the 12 months before enrollment. Therefore, the period covered by this sub-study analysis is February 2010 through February 2014. We collected the baseline data using two methods: a patient-completed survey given to the patient/parent on the date of enrollment, and medical record review of all the patients’ visits to the enrollment site for the 12 months before enrollment, not including the enrollment visit.

### Patients and practices

**Figure 1:**
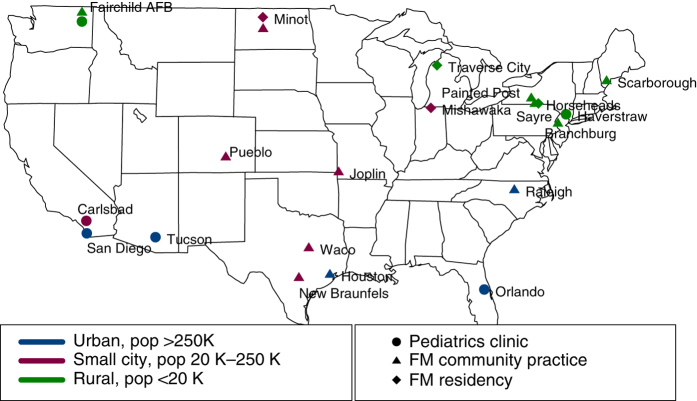
Practice sites by practice type and community size.

Patients were enrolled from the 22 enrolled family medicine and paediatrics community practices. The practices were selected from primary care practices that were members of one or more practice-based research network, attempting to include sites from several regions of the US (SouthEast, NorthEast, MidWest, SouthWest and West Coast) and from rural areas, small- to medium-sized cities and large urban areas. (See designation on the map, Figure 1.) Patients were identified as having persistent asthma and identified by an asthma ICD-9 diagnostic code plus a current prescription for one or more daily maintenance medications from the practice’s electronic health record or as patients meeting these characteristics visited the practice for asthma-related concerns. Of the patients offered enrollment, over 90% accepted.

### Data collection

The baseline enrollment survey collected extensive patient/parent-reported information on the following: patient’s personal characteristics; asthma-related information—sources of asthma care; number of visits to the emergency department hospital; or receipt of oral or intramuscular steroids for asthma in the previous 6 months, as well as difficulty obtaining asthma care in the previous year. A measure of the enrolled patients’ level of current asthma control was collected using the Asthma APGAR.^22,23^ The Asthma APGAR asks three questions about the frequency of activities that were missed or modified because of breathing problems, the frequency of daytime asthma symptoms and the frequency of night-time asthma symptoms over the previous weeks. An Asthma APGAR score of >2 is comparable to an Asthma Control Test score of <20.^23,24^ In addition to a ‘control score’, the Asthma APGAR asks questions about asthma triggers, use and adherence to asthma medications and the patient’s assessment of the impact of their asthma medications. The responses are linked to a care algorithm recommending next steps of care. For the baseline period, the practices did not have access to the Asthma APGAR. (Asthma APGAR is displayed in the Supplementary Figure.)

The patient’s age, sex, race/ethnicity, level of obesity, smoking status, estimated weekly activity levels, exposure to secondhand smoke and household income were all patient reported. Obesity was assessed on the basis of the patient’s body mass index (BMI, height and weight) measured and documented in the patient’s medical record during a visit in the baseline year. We required that both height and weight be measured (not patient estimate or report) at the same visit in children and adolescents and within the baseline year for adults, as adults are unlikely to gain height. The BMI for children aged 5 through 18 years is reported as the percentile on standard age-specific growth curves and summarised as underweight (<5%), normal weight (5 to 85%), overweight (>85 to 95%) and obese (>95%).^25,26^ For adults, we used the US Center for Disease Control and Prevention (CDC) recommended cut-offs for underweight (BMI <18.5), normal weight (BMI 18.5–24.9), overweight (25.0–29.9), obese (30.0–35.0) and morbidly obese (BMI >35.0).^27,28^

The self-reported data were collected using previously developed or validated questions whenever possible. Smoking status, race/ethnicity and household income were collected using questions from the Behavioral Risk Factor Survey,^29^ the National Health Interview Survey^30^ or Medical Expenditure Panel Survey.^31^ When we found no standardised or validated question, we developed questions specifically for this study. Activity frequency was asked on a daily basis, which allowed collapse into longer periods. The levels of activity queried (moderate or strenuous) were adapted from the 2000 International Physical Activity Questionnaire,^32^ and results were compared with the 2010 CDC recommendations for physical activity.^33,34^ The questions on secondhand smoke exposure were designed to address the sights where children, adolescents and adults spend significant portions of time that are not regulated by indoor smoking laws: e.g., home and cars. The questions included in the baseline survey are in Supplementary Table 1.

We chose to present results within age groupings similar to those used in the US national asthma guidelines—e.g., 5 through 11 years, 12 through 18 years and 19 years and older, and labelled the groups as children, tweens and adults, respectively.^35^

The asthma control outcome is based on the patient/parent’s completed Asthma APGAR score, with a score of >2 considered to be inadequate control.^22,23^ To compare patient-perceived asthma control with that from a validated objective metric, we used the final question from the Asthma Control Test, which asks patients to report their perceived level of asthma control.^24^

Overall, 1,176 patients were enrolled in the Asthma Tools study between 2011 and 2014. Of the enrolled patients, 901 (77%) returned a useable baseline survey; e.g., responses were completed to over 90% of survey items. The rates of return varied among the enrolled patients from 76% in children to 73% in tweens and 67% in men to 83% in women (*P*=0.03). Across all age groups, male patients were less likely than female patients to return the survey, although the differences were only significant for the adults. Among the adults, younger individuals (*P*<0.0001) and those with higher BMIs (*P*<0.05) were also less likely to return baseline surveys.

### Data analysis

Patient characteristics were tabulated, and percentages were computed based on the individuals responding to the item. Characteristics were compared using Wilcoxon rank-sum tests for numeric data and Chi-squared tests for frequencies. Logistic regression was used to identify characteristics associated with asthma control, as determined by Asthma APGAR >2 and for asthma exacerbations determined by self-reported emergency department (ED) or hospital visits or steroid bursts for asthma. For multivariable modelling, a step-down procedure was used, starting with all variables and removing those with the highest *P* values first. All analyses were completed in S-Plus v. 7.0.6 (Tibco, Boston, MA, USA).

## Results

### Demographics of the primary care asthma sample

Table 1 summarises the demographic information for the 901 primary care patients included in this sub-study. Enrollment rates by sex reflect the known higher prevalence of asthma in boys, reversing to more women than men in adulthood. The population is racially and ethnically diverse with 20% or more Hispanic individuals in each age group but decreasing numbers of nonwhite individuals across increasing age groups. The annual family income was low for these patients with 42% of the children, 31% of tweens and 33% of adults living in a household with annual household incomes ⩽$25,000, which is approximately the 2011 Federal poverty level for a household of three or more.^36^

Across all age groups, more than 35% of the asthma patients had BMIs considered to be in the overweight or obese range. It means that 35% of the children and 46% of the tweens had BMIs at or above the 85 percentile of weight for all similarly aged children or tweens of the same sex. Overall, 46% of the adults had a BMI of 25 or greater. Exposure to tobacco smoke was common among all enrollees, with more than 1 in 8 adults reporting themselves as smokers with daily secondhand smoke exposure in 1 in 8 children, 1 in 6 tweens and 1 in 4 adults.

Self- or parent-reported activity decreased with age. Although 30% of children and 28% of tweens met or exceeded the CDC’s recommendation for levels of weekly activity of 15 or more minutes of moderate activity 5 or more days a week, only 9% of adults met the recommended 15 or more minutes of moderate activity 5 or more days per week.^33,34^ Girls and women were less likely than boys or men to do at least 15 or more minutes of strenuous activity 5 or more days per week.

### Patient assessments versus objective assessment of asthma control and quality of life

Almost half of this cohort had inadequate asthma control according to the patient-completed Asthma APGAR.^22,23^ Higher rates of inadequate control were reported in tween girls compared with tween boys and women compared with men (Table 2). Despite high rates of objective assessment of inadequate asthma control, only about 10% of tweens and 15% of adults said their asthma was not in control, as defined by the final question of Asthma Control Test.^23,24^ The Childhood Asthma Control Test does not include this question.

Asthma-related ED or urgent care visits in the past 6 months were common in all ages but highest in children (5- to 11-year-old), in whom almost 1 in 4 reported that they made such a visit. The rates dropped in tween (12- to 18-year-old) boys, but rates remained high among tween girls. The sex differences in rates of ED visits continued into adulthood (Table 2). Rates of frequent ED or urgent care visits, e.g., three or more in the previous 6 months, were more common in female patients of all ages compared with male patients, but did decline with increasing age (7.8% of children, 3.4% of tweens and 3.6% of adults). Hospitalisations also demonstrated the same sex differential as ED visits, across all ages (Table 2).

Even with the high rates of urgent care and ED visits, over 1 in 10 tweens and 1 in 5 adults reported inability to obtain needed asthma care at least once in the baseline year. We did not ask the question for children aged 5–11 years.

### Logistic regression to identify associations with asthma control and exacerbations

Several individual socio-demographic and behavioural factors were associated with out-of-control asthma, and the factors differed by age group. Univariable analysis results are in Supplementary Table 2. Among children, out of control asthma was associated with annual family income <$50,000 and being overweight. No factors were found to be significant in tween groups aged 12 through 18 years, perhaps because of our limited sample of tweens. In adults, female gender, Black race, annual family income less than $50,000, less than 15  min of activity 5 or more days a week, smoking and obesity were each individually associated with out-of-control asthma. In multivariable modelling, adding in all the factors that were individually associated with out-of-control asthma, only annual family income <$50,000 was significant for children, although obesity showed a trend towards significance (*P*=0.06) and for adults family income <$50,000, smoking and obesity remained jointly significant (Table 3). Among adults, the likelihood of having out-of-control asthma increased with increasing BMI across the entire spectrum of weight from underweight to morbid obesity (*P*=0.005 for a linear trend).

The factors associated with having one or more asthma exacerbations during the previous 6 months were slightly different from those for out-of-control asthma. In children, only annual family income of <$50,000 was significant in both univariable and multivariable modelling. For the tween group, both obesity and Black race were significant in univariable and multivariable modelling. For adults 19 years and older, Black race, annual family income <$50,000, obesity and smoking were each significantly associated with asthma exacerbations. Race, obesity and smoking remained significant in multivariable logistic regression in adults (Table 4).

## Discussion

### Main findings

This group of children, tweens and adults have multiple adverse health and asthma risk factors. They are commonly exposed to tobacco smoke, often overweight, have modest levels of physical activity, limited family incomes, significant asthma-related health-care utilisation of urgent, emergent and hospital care, high rates of out-of-control asthma and a significant self-reported unmet need for asthma care. These characteristics suggest multiple opportunities for intervention and highlight potential barriers that family physicians and paediatricians face in providing asthma care to their patients.

### Interpretation of findings in relation to previously published work

Among our group of patients, smoking was significantly associated with both out-of-control asthma and asthma exacerbations among the adults. This provides further support for the need to address smoking at each visit, as repeated attempts for smoking cessation supported by combination therapy can reduce smoking rates significantly.^37,38^ The self-reported rates of tobacco use of these adults are similar to the overall US population for women (16.0% vs. 15.8% for US) but significantly lower among the men (13.3% vs. 20.5% for US, *P*<0.01).^39^ Whether this result reflects men’s enhanced smoking abstinence in the face of asthma or differences in reporting choices is unknown, but worth further exploration in other populations of adults with asthma. The self-reported smoking rates of tweens are very low and may represent reluctance of the tweens to report smoking on a survey reviewed by their parents.

Although secondhand smoke exposure did not rise to the level of statistical significance in relation to poor asthma outcomes, tobacco smoke is a well-recognised trigger for asthma symptoms and exacerbations.^17–19^ Exposure to any secondhand smoke was common among these patients varying from about 1 in 3 children to over half of adults, with more than 1 in 10 of these individuals exposed to daily secondhand smoke. However, physicians and other clinicians do not often assess or at least document environmental tobacco smoke exposure.^40,41^ Any visit related to asthma, especially those for an exacerbation or review of asthma, should include a query, documentation and offer to support parents and others in smoking-cessation efforts.^17–19^ Every asthma visit represents an intervention opportunity.

At every age, being overweight or obese was associated with adverse asthma outcomes: having out-of-control asthma in children and adults, and experiencing asthma exacerbations in tweens and adults. Compared with the US obesity rates reported from the CDC’s National Health and Nutrition Examination Survey, these children and tweens with asthma were 1.2–1.3 times more likely to be obese (>95% of expected weight for age).^26^ The problem was even more pronounced in adults in whom obesity and morbid obesity rates were 1.5 times that of the US adult population.^27,28^ Other studies have also linked obesity with adverse asthma outcomes, including poorer control and increased asthma severity.^42^ Our data highlight an important opportunity for family physicians and paediatricians to discuss the impact of weight, eating and activity choices on adverse asthma outcomes that can be disruptive and expensive for families.^3,4,6^ Unfortunately, limited data exist to recommend simple solutions, with most weight management programmes requiring intensive long-term intervention.^43,44^

Among this group of people with asthma, activity levels were reported to be moderate to low and to decrease to very low levels in tween girls and women. Up to 20% of the children and tweens and over 33% of women reported no days of even 15 min of activity in a week. Current US CDC recommendations for the general health of children, adolescents and adults include at least 60 min of moderate or more strenuous aerobic activity each day with added muscle strengthening and bone strengthening pursuits.^33,34^ Primary care physicians and other clinicians have an opportunity to discuss the impact of activity on asthma and weight management.^16^

Patients’ willingness to engage in discussions related to changing behaviours may be based on their perceptions of health (e.g., asthma control) and personal goals.^45^ As has been reported previously,^46^ this group of parents and patients also appeared to overestimate their asthma control. This may represent a personal preference related to the perceived burden versus benefit of behaviours or lifestyle choices that could improve asthma control. Conversely, it may represent a misunderstanding of what has to be accepted as ‘normal’ for a person with asthma. Awareness of the gap between patient/parent and health professionals’ assessments of asthma status may represent another important opportunity for addressing both medication use and lifestyle changes to address factors adversely affecting asthma including obesity, smoking and activity.

### Strengths and limitations of this study

The strengths of this study include the large sample size, the inclusion of patients from a wide diversity of regions of the United States, and multiple practices. We collected data on several health-related conditions that are seldom addressed in pragmatic asthma trials including secondhand smoke exposure, activity and obesity. Our study also has several limitations including responder bias. As reported previously,^7^ the responders to the initial survey differed from the nonresponders in some characteristics that we could identify from the medical record reviews, including age, gender and BMI, with the nonresponders more likely to be men, younger and to have a BMI in the overweight or obese range. It is not clear how age and gender might affect the factors we found to be associated with poorer asthma outcomes. However, it is possible that the decreased responder rate for obese individuals may have resulted in an underestimation of the impact of obesity on the outcomes of interest. Some of the factors reported are not modifiable in the usual health-care setting but could be used to alert health-care professionals to the increased likelihood of poor asthma outcomes and to search for modifiable factors such as inability to buy healthier foods, avoid smoke exposure or the lack of a safe place to do physical activities.

### Implications for future research, policy and practice

Future studies that address pragmatic approaches to improved asthma outcomes need to go beyond medication-based asthma management to address other modifiable factors and lifestyle choices. Specifically, prospective studies to further establish causality between lifestyle choices and asthma outcomes are required, as are clinical trials to demonstrate the ability of interventions to increase exercise and reduce weight and smoke exposure to improve asthma control. Primary care is the appropriate site to encompass approaches to most of the patient’s health-care needs, and these data stress the continuing need to address obesity, activity, secondhand smoke exposure and smoking in asthma management.

### Conclusions

Among this sample of primary care patients with asthma, inadequate asthma control and risk of exacerbation were associated with factors for which interventions are possible: smoking, exposure to secondhand smoke, low levels of physical activity and obesity. These findings suggest that primary care health professionals consider these potentially modifiable elements of asthma management during each visit.

## Figures and Tables

**Table 1:** Demographic and self-reported behaviors

*Characteristics by age group*	*Enrolled and returned baseline survey N=893*		
*Children (5–11 years)*	*All*	*Boys*	*Girls*
	*N=216*	*N=137*	*N=79*
Age in years: mean	8.4	8.5	8.3
	%	%	%
			
*Race/ethnicity*			
White	66.7	64.7	67.1
Black	26.7	26.3	25.3
Native American	0.3	0.8	0
Asian	4.0	5.3	5.1
Other/none	2.3	3.0	2.5
Hispanic	24.7	26.3	29.1
			
*Family income per year*			
<$10,000	15.9	17.0	13.9
$10,000 to $24,999	21.0	20.7	21.5
$25,000 to $49,999	20.1	17.1	25.3
⩾$50,000	39.7	40.8	38.0
Not reported	3.3	4.4	1.3
			
*BMI*			
% Obese	21.4	16.9	29.0
% Overweight	14.0	14.6	13.1
% Healthy	56.5	59.6	51.4
% Underweight	3.5	5.1	0.9
% Not available	4.6	3.9	5.6
			
*Smoking*			
% Any secondhand smoke	35.7	35.6	35.8
5–7 Days per week ever	21.1	21.8	20.3
5–7 Days per week now	12.7	12.8	12.7
			
*Activity level*			
% Doing moderate activity			
⩾3 Times a week, 15 min	64.4	65.0	63.3
⩾5 Times a week, 15 min	39.4	39.4	39.2
% Doing strenuous activity			
⩾3 Times a week, 15 min	58.3	52.0	62.0
⩾5 Times a week, 15 min	29.6	36.5	17.7
			
Parent-reported use of influenza vaccine (% always or usually)	69.4	67.9	72.2
Allergy testing ever, % yes	43.3	45.9	38.0
Needed asthma care and could not get it	6.1	7.5	3.9
			
*Tweens (12–18 years)*	*All*	*Boys*	*Girls*
	*N=153*	*N=83*	*N=70*
Age in years: mean^a^	15.2	14.7	15.8
			
*Race/ethnicity*			
White	78.4	75.6	82.1
Black	14.4	20.0	11.9
Native American	1.3	2.3	0
Asian	2.6	3.5	1.5
Other/none	3.7	2.3	4.5
Hispanic	24.8	22.1	28.4
			
*Family income per year*			
<$10,000	13.8	8.2	20.9
$10,000 to $24,999^a^	17.1	18.8	14.9
$25,000 to $49,999	16.4	15.3	17.9
⩾$50,000	45.4	49.5	40.3
Not reported	7.2	8.2	6.0
			
*BMI*			
% Obese	27.5	28.3	26.5
% Overweight	19.0	14.2	24.5
% Healthy	43.1	46.0	39.8
% Underweight	3.8	6.2	1.0
% Not available in 1 year	6.6	5.3	8.2
			
*Smoking –*			
Primary	1.3	1.3	1.5
Secondhand smoke (any)	54.1	49.4	59.7
5–7 Days per week ever	24.8	24.4	25.4
5–7 Days per week now	17.0	18.6	14.9
			
*Activity level*			
% Doing moderate activity			
⩾3 times a week, 15 min	58.2	55.8	61.2
⩾5 times a week, 15 min	30.7	30.2	31.3
% Doing strenuous activity			
⩾3 Times a week, 15 min	51.0	53.5	47.8
⩾5 Times a week, 15 min	28.1	30.2	25.4
			
Self-reported use of influenza vaccine (% always or usually)	64.7	65.0	64.2
Allergy testing ever, % yes	44.7	44.2	44.8
Needed asthma care and could not get it	6.8	6.3	7.6
			
*Adults >18 years (19–60 years)*	*All*	*Men*	*Women*
	*N=533*	*N=128*	*N=405*
Age in years: mean	40.6	39.8	40.9
			
*Race/ethnicity*			
White	89.3	91.4	88.6
Black	7.9	3.9	8.4
Native American	1.0	2.3	1.0
Asian	1.0	1.6	1.0
Other/none	1.0	0.8	1.0
Hispanic	3.9	5.5	4.2
			
*Family income/year*			
<$10,000	16.5	10.9	18.3
$10,000 to $24,999	16.5	10.9	18.3
$25,000 to $49,999	19.2	20.3	18.7
⩾$50,000^a^	45.9	56.3	42.7
Not reported	1.9	1.6	2.0
*BMI (mean and range)*	32.6 (12–71)	30.9 (12–52)	33.1 (19–71)
% Morbidly obese	16.8	8.6	19.4
% Obese	33.6	32.0	34.1
% Overweight	25.2	34.4	22.4
% Normal weight	15.0	7.8	17.2
% Under weight	0.4	1.6	0
% Not reported	9.0	15.6	6.9
			
*Smoking*			
Primary	15.4	13.3	16.0
Secondhand smoke (any)	60.7	60.3	60.8
5–7 Days per week ever	64.2	62.5	64.7
5–7 Days per week now	22.5	18.8	23.7
			
*Activity level*			
% Doing moderate activity			
⩾3 Times a week, 15 min	39.8	43.0	38.8
⩾5 Times a week, 15 min	18.5	23.4	17.5
% Doing strenuous activity			
⩾3 Times a week, 15 min	24.0	37.5	19.8
⩾5 Times a week, 15 min	9.0	15.6	6.9
			
Self-reported use of influenza vaccine (% always or usually)	58.5	57.0	59.0
Allergy testing ever, % yes	57.2	63.3	54.6
Needed asthma care and could not get it	20.8	15.7	21.9

a Those with statistical difference in row frequencies (*P*<0.05).

**Table 2:** Self-reported asthma outcomes by age group

*Asthma-related outcomes by age group*	*Enrolled and returned baseline survey* N*=893*		
*Children (5–11)*	*All* N*=214*	*Boys* N*=137*	*Girls* N*=79*
% Asthma out of control^+^	56.0	58.5	51.9
			
*% With asthma visits to:*			
ED/urgent	27.3	29.9	23.1
Hospital	6.2	3.9	9.1
Steroid burst ⩽1 previous year	32.7	30.4	36.7
Steroid burst >1 previous year	13.1	10.1	14.8
% With an ‘exacerbation’	34.3	38.3	27.8
AQLQ—mean	5.2	5.1	5.3
*Tweens (12–18)*	*All* *N* *=153*	*Boys* *N* *=83*	*Girls* *N* *=70*
% Asthma out of control^+,^^a^	52.0	45.9	59.7
% Self-reported asthma out of control	10.0	10.1	11.3
			
*% With asthma visits to:*			
ED/urgent	18.5	15.2	22.3
Hospital	2.8	1.3	4.6
Steroid burst ⩽1 previous year	18.4	17.6	19.4
Steroid burst >1 previous year	6.5	5.9	7.4
% With an ‘exacerbation’	14.1	13.0	15.4
AQLQ—mean	5.2	5.3	5.0
*Adults >18 years (19–60)*	*All* *N* *=533*	*Men* *N* *=128*	*Women* *N* *=405*
% Asthma out of control^+,^^a^	61.2	53.1	63.7
% Out of control by self report	15.9	14.1	16.5
			
*% With asthma visits to:*			
ED/urgent	18.8	12.1	19.1
Hospital	3.5	1.6	4.0
Steroid burst ⩽1 previous year	20.0	18.0	20.6
Steroid burst >1 previous year	9.0	7.8	9.3
% With an ‘exacerbation’	23.2	17.9	24.9
AQLQ—mean	5.0	5.3	4.9

+Asthma APGAR >2.

a Denotes those with statistical difference in row frequencies (*P*<0.05).

**Table 3:** Factors associated with out-of-control asthma

*By age group*	*Factor*	*Odds ratio (CI 95%)*	P-*value*
*5–11 years*			
Univariable	Obesity^a^	2.3 (1.1–4.7)	0.03
	Family income^b^<$50,000	1.5 (1.1,2.0)	0.007
Multivariable	Family income <$50,000	1.5 (1.1,2.0)	0.007
*12–18 Years*	No factors are significant		
*19+ Years*			
Univariable	Smoking^c^	2.3 (1.5,3.5)	<0.001
	Family income <$50,000	2.1 (1.5,3.0)	<0.001
	Race (Blacks)^d^	2.2 (1.03,4.8)	0.02
	Morbid obesity	1.5 (1.1,2.2)	0.008
	Less activity^e^	1.5 (1.1, 2.2)	0.01
	Gender (female)	1.6 (1.04,2.3)	0.02
Multivariable	Smoking	1.7 (1.1,2.7)	0.01
	Morbid obesity	1.7 (1.1,3.0)	0.02
	Family income <$50,000	1.6 (1.1,2.4)	0.01

Variables considered are as follows: age, gender, race, income, activity, flu vaccination, smoke exposure and obesity. Variables were fit singly (univariate modelling), and then a stepwise procedure examined all the variables together.

a The definition for obesity was appropriate to the age group considered and varied for those <19 and adults.^37,38^ Non-obese was the reference group.

b Family income was self reported as total annual family income with reference group income of ⩾$50,000/year.

c Smoking was self reported; current smoking status and nonsmoking was the reference group.

d Race was self-reported race. White was the reference group.

e Activity was self reported as the number of times per week with 15 min or more of moderate and 15 min or more of strenuous activity (examples given). Higher levels of activity, at least 15 min of moderate activity five or more times per week or 15 or more minutes of strenuous activity two or more times per week.

**Table 4:** Factors associated with ⩾1 asthma exacerbations in previous 6 months

*By age group*	*Factor*	*Odds ratio (CI 95%)*	P-*value*
*5–11 Years*			
Univariable	Family income^a^ <$50,000	1.5 (1.1,2.0)	0.01
Multivariable	Family income <$50,000	1.5 (1.1, 2.0)	0.01
			
*12–18 Years*			
Univariable	Obesity^b^	2.9 (1.6,5.1)	<0.0001
	Race^c^	0.3 (0.1,0.9)	0.03
Multivariable	Obesity	3.0 (1.7,5.3)	<0.0001
	Race (Black)	0.3 (0.1,0.9)	0.03
			
*19+ Years*			
Univariable	Smoking^d^	1.3 (1.04,1.6)	<0.0001
	Family income <$50,000	1.4 (1.1,1.7)	0.003
	Race (Blacks)	2.4 (1.2,4.7)	0.01
	Morbid obesity	1.7 (1.1,2.6)	0.02
Multivariable	Smoking	1.3 (1.04,1.6)	0.02
	Morbid obesity	1.7 (1.1,2.6)	0.02
	Race (Black)	4.0 (2.0,8.0)	<0.001

Variables considered are as follows: age, gender, race, income, activity, flu vaccination, smoke exposure and obesity. Variables were fit singly (univariate modelling), and then a stepwise procedure examined all the variables together.

a Family income was self reported as total annual family income with reference group income of $50,000 or more per year.

b The definition for obesity was appropriate to the age group considered and varied for those <19 and adults.^37,38^ Non-obese was the reference group.

c Race was self-reported race. White was the reference group.

d Smoking was self reported; current smoking status and nonsmoking was the reference group.
