# Supplementary material for: Obesity, low levels of physical activity and smoking present opportunities for primary care asthma interventions: an analysis of baseline data from The Asthma Tools Study
Source: NPJ Prim Care Respir Med. 2015 Oct 1;25:15058–. doi: 10.1038/npjpcrm.2015.58 (PMC4590305; doi:10.1038/npjpcrm.2015.58)
Supplement: Supplementary Table 1 [file npjpcrm201558-s1.doc]

**Supplementary Table 1: Questions about activity and smoking.**

| **During the *last 7-day period (a week),* how many times on average did you do the following kinds of exercise for more than 15 minutes?** | | | | | | | | | |
| --- | --- | --- | --- | --- | --- | --- | --- | --- | --- |
|  | **None** | **1 time** | **2 times** | **3**  **times** | **4 times** | **5 times** | **6 times** | **7 times** | **8 times**  **or more** |
| **Strenuous exercise (heart beats rapidly)** (i.e., running, jogging, vigorous swimming, vigorous long-distance bicycling, hockey, basketball, cross-country skiing, soccer) |  |  |  |  |  |  |  |  |  |
| **Moderate exercise (not exhausting)**  (i.e., fast walking, easy swimming, alpine skiing, popular and folk dancing, tennis, easy bicycling, baseball, volleyball) |  |  |  |  |  |  |  |  |  |
| **Mild exercise (minimal effort)**  (i.e., easy walking, archery, bowling, horseshoes, golf, snowmobiling) |  |  |  |  |  |  |  |  |  |

| **I currently smoke cigarettes or a pipe daily.*** | **Yes** | **No** |
| --- | --- | --- |
|  |  |

| **In the past *6 months* how often have the following happened?** | **Not at all** | **Occasionally** | **Sometimes** | **Frequently** |
| --- | --- | --- | --- | --- |
| **People smoked in my house** |  |  |  |  |
| **People smoked in the car that I rode in** |  |  |  |  |
| **People smoked in my work place** |  |  |  |  |
| **I went to friends’ houses that had a lot of smoke** |  |  |  |  |
| **I went to places like restaurants or bars that had a lot of smoke** |  |  |  |  |
| **We used an indoor wood fireplace** |  |  |  |  |

***This question was asked only in those 12 years and older**
