# Supplementary material for: Obesity, low levels of physical activity and smoking present opportunities for primary care asthma interventions: an analysis of baseline data from The Asthma Tools Study
Source: NPJ Prim Care Respir Med. 2015 Oct 1;25:15058–. doi: 10.1038/npjpcrm.2015.58 (PMC4590305; doi:10.1038/npjpcrm.2015.58)
Supplement: Supplementary Table 2 [file npjpcrm201558-s2.doc]

**Supplementary Table 2. Univariable associations of variables with patient outcomes.**

|  | Asthma out of control | | At least one exacerbation | |
| --- | --- | --- | --- | --- |
|  | OR (95% CI) | P-value | OR (95% CI) | P-value |
| Age 5-11 |  |  |  |  |
| Age > median | 1.10 (0.84, 1.44) | .50 | 1.45 (1.10, 1.90) | .01 |
| Sex (Female) | 1.14 (0.86, 1.51) | .35 | 1.08 (0.82, 1.42) | .58 |
| Race (Black) | 1.69 (0.89, 3.20) | .11 | 1.06 (0.57, 1.97) | .86 |
| Income < $50K | 1.48 (1.12, 1.96) | .01 | 1.47 (1.09, 1.99) | .01 |
| Reduced activity level | 1.06 (0.78, 1.45) | .70 | 1.02 (0.74, 1.40) | .92 |
| Flu vaccination | 1.71 (0.91, 3.22) | .10 | 1.06 (0.56, 1.99) | .86 |
| Smoking exposure | 1.07 (0.71, 1.63) | .74 | 1.30 (0.86, 1.97) | .21 |
| Obesity | 2.3 (1.07, 4.65) | .03 | 1.08 (0.62, 1.89) | .78 |
| Age 12-18 |  |  |  |  |
| Age > median | 1.33 (0.69, 2.53) | .39 | 1.35 (0.90, 2.02) | .15 |
| Sex (Female) | 1.72 (0.89, 3.30) | .11 | 1.04 (0.70, 1.54) | .86 |
| Race (Black) | 1.25 (0.49, 3.18) | .64 | 0.34 (0.08, 0.89) | .03 |
| Income > $50K | 1.29 (0.66, 2.51) | .46 | 1.24 (0.79, 1.95) | .35 |
| Reduced activity level | 1.20 (0.60, 2.38) | .61 | 1.10 (0.69,1.74) | .69 |
| Flu vaccination | 1.19 (0.59, 2.40) | .64 | 1.58 (0.58, 4.33) | .38 |
| Smoking exposure | 1.04 (0.52, 2.08) | .91 | 1.03 (0.64, 1.64) | .91 |
| Obesity | 2.75 (0.28, 27.06) | .39 | 2.88 (1.56, 5.06) | <.001 |
| Age 19+ |  |  |  |  |
| Age < median | 1.00 (0.71, 1.42) | .99 | 1.89 (1.27, 2.83) | .002 |
| Sex (Female) | 1.58 (1.04, 2.34) | .02 | 1.07 (0.68, 1.66) | .78 |
| Race (Black) | 2.23 (1.03,4.79) | .02 | 2.36 (1.18, 4.68) | .01 |
| Income < $50K | 2.05 (1.46, 2.95) | <.001 | 1.43 (1.14,1.73) | .003 |
| Reduced activity level | 1.55 (1.05, 2.15) | .01 | 1.31 (0.87, 1.97) | .20 |
| Flu vaccination | 1.09 (0.76, 1.56) | .63 | 1.31 (0.87, 1.98) | .20 |
| Smoking exposure | 2.25 (.1.47, 3.45) | <.001 | 1.34 (1.04, 1.63) | .02 |
| Morbid Obesity | 1.54 (1.07, 2.24) | .01 | 1.74 (1.09, 2.56) | .02 |
