# Supplementary material for: Obesity, low levels of physical activity and smoking present opportunities for primary care asthma interventions: an analysis of baseline data from The Asthma Tools Study
Source: NPJ Prim Care Respir Med. 2015 Oct 1;25:15058–. doi: 10.1038/npjpcrm.2015.58 (PMC4590305; doi:10.1038/npjpcrm.2015.58)
Supplement: Supplementary Figure 1 [file npjpcrm201558-s3.pdf]

Supplementary Figure 1

**A**= Activities<br>**P**=Persistent<br>**G**=triGGers<br>**A**= Asthma medications<br>**R**= Response to therapy	**P**=Asthma Plan<br>**L**=Lung function<br>**U**=Use of inhaler<br>**S**=Steroids

APGAR PLUS

Please circle your answers:

A

1. In the past 2 weeks, how many times did any breathing problems (such as asthma) interfere with your ACTIVITIES or activities you wanted to do?

Never 1 – 2 times 3 or more times

P

2. How many DAYS in the past 2 weeks did you have shortness of breath, wheezing, chest tightness, cough or felt you should use your rescue inhaler?

None 1 - 2 DAYS 3 or more DAYS

3. How many NIGHTS in the past 2 weeks did you wake up or have trouble sleeping due to coughing, shortness of breath, wheezing, chest tightness or get up to use your rescue medication?

None 1 - 2 NIGHTS 3 or more NIGHTS

G

4. Do you know what makes your breathing problems or asthma worse?

Yes No Unsure

- **Please circle things that make your breathing problems or asthma worse:**

  Cigarettes Smoke Cold Air Colds Exercise Dust Dust Mites

  Trees Flowers Cats Dogs Mold Other: ____________________

- **Can you avoid the things that make your breathing problems or asthma worse?**

  Seldom Sometimes Most of the time

A

5. List or describe medications you've taken for breathing problems or asthma in the past 2 weeks: Remember you may use Nasal, Oral, or Inhaler medications.

Breathing or Asthma Medication	When taken?		Reasons for taking medication:	Reasons for not taking medication:
	☐ Daily	☐ As needed		
	☐ Daily	☐ As needed		
	☐ Daily	☐ As needed		
	☐ Daily	☐ As needed		

R

6. When I use my breathing or asthma medicines I feel:

Worse No Different A Little Better A Lot Better
